# Comparison of Porcine Epidemic Diarrhea Viruses from Germany and the United States, 2014

**DOI:** 10.3201/eid2103.141165

**Published:** 2015-03

**Authors:** Dennis Hanke, Maria Jenckel, Anja Petrov, Mathias Ritzmann, Julia Stadler, Valerij Akimkin, Sandra Blome, Anne Pohlmann, Horst Schirrmeier, Martin Beer, Dirk Höper

**Affiliations:** Friedrich-Loeffler-Institut, Greifswald–Insel Riems, Germany (D. Hanke, M. Jenckel, A. Petrov, S. Blome, A. Pohlmann, H. Schirrmeier, M. Beer, D. Höper);; Ludwig-Maximilians-University Munich, Oberschleissheim, Germany (M. Ritzmann, J. Stadler);; Chemical and Veterinary Investigations Office Stuttgart, Fellbach, Germany (V. Akimkin)

**Keywords:** porcine epidemic diarrhea virus, German case report, new variant, European isolates, next-generation sequencing, viruses, Germany, Europe

## Abstract

Since 2013, highly virulent porcine epidemic diarrhea virus has caused considerable economic losses in the United States. To determine the relation of US strains to those recently causing disease in Germany, we compared genomes and found that the strain from Germany is closely related to variants in the United States.

Porcine epidemic diarrhea (PED) is an acute and highly contagious enteric disease of swine that results in severe enteritis, diarrhea, vomiting, and dehydration (*1*). Porcine epidemic diarrhea virus (PEDV), the causative agent, is an enveloped, positive single-stranded RNA virus that belongs to the family *Coronaviridae*, genus *Alphacoronavirus* (*2*).

The disease was first recognized in Europe in 1971 and has thereafter caused high economic losses, particularly in Asia. In May 2013, a highly virulent PEDV variant emerged in the United States; explosive epidemics on swine farms affected pigs of all ages, resulting in a mortality rate of up to 95% among suckling pigs (*3*). Since then, outbreaks have occurred in 30 US states (*4*), causing very high economic losses, and the disease threatens to spread. The involved viruses cluster together with isolates from China from 2011 and 2012 (*5*). Apart from these highly virulent strains, a PEDV variant from the United States (strain OH851) that affected sows instead of younger animals and caused milder disease was recently described (*6*).

The effect of PED in the United States has unsettled pig farmers and veterinarians worldwide; studies have been recently initiated to elucidate the situation in Europe. Despite the history of PED outbreaks in Europe, little is known about currently circulating virus strains and their effect; information about the phylogeny of recent strains and their relation to the outbreak strain in the United States is lacking.

We report a case of PED that occurred on a swine-fattening farm in Germany in May 2014. The causative virus was fully characterized by using conventional methods and next-generation sequencing. We analyzed the resulting full-length genomes and compared them with those of the strains circulating in the United States and Asia to elucidate possible relationships.

## The Study

**Figure 1:**
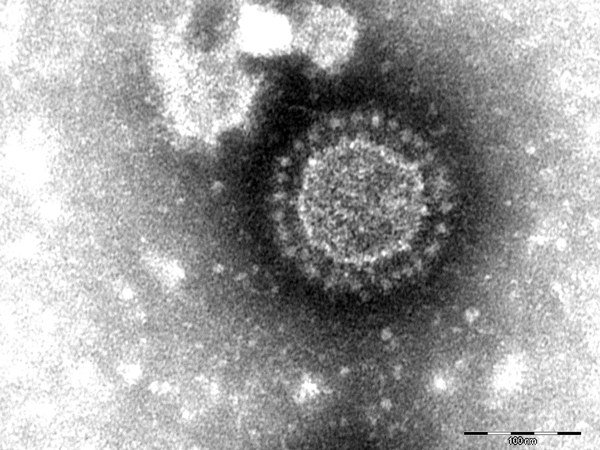
Porcine epidemic diarrhea virus particles seen by negative-stain electron microscopy of fecal samples. Negative staining with 1% phosphotungstic acid. Scale bar indicates 100 nm.

In May 2014, a pig fattening farm in southern Germany (Federal State of Baden-Wuerttemberg) that continuously houses ≈1,400 fattening pigs reported watery diarrhea in pigs in all age groups (feeders to slaughter animals). The first cases occurred after new feeder pigs from a large piglet producer were brought onto the farm. The incoming animals showed diarrhea 2 days after arrival. Within 1 day thereafter, the disease had spread to pigs in all other age groups. Clinical signs were present for at least 1 week; ≈20 pigs died. Fecal samples from diseased pigs were submitted to the regional laboratory for diagnosis, and coronaviruses were detected by electron microscopy (Figure 1). Additionally, 3 pigs with catarrhal enteritis were euthanized; postmortem examination at the regional laboratory confirmed coronavirus infection in all 3 animals. 

Subsequently, PED was diagnosed in a private laboratory (IVD GmbH, Hannover, Germany) by use of a published multiplex reverse transcription quantitative PCR (*7*). Selected positive samples were submitted to the Friedrich-Loeffler-Institut, Isle of Riems, Germany, for confirmation and further virus characterization. At this institution, 2 fecal samples with high genome load, as determined by 2 independent, published (*8*,*9*) reverse transcription quantitative PCRs selective for PEDV nucleocapsid (N) and spike (S) genes, were chosen for routine virologic testing and next-generation sequencing. Sequencing and data analyses were performed as previously described (*10*). The phylogenetic tree, based on full-length genomes, was constructed by using PhyML (*11*) in the Geneious software suite (http://www.geneious.com/) with a generalized time reversible substitution model, and the tree was supported by 1,000 bootstrapping replicates.

Virus isolates were obtained after inoculation of cells of different permanent cell lines (pig kidney [PK]-15 and Vero) with clinical material from the pigs. Sequencing of nucleic acids isolated directly from diagnostic samples PEDV/GER/L00719/2014 and PEDV/GER/L00721/2014 resulted in 2 viral genomes (Table) encompassing all typical PEDV coding sequences. Each sequence encodes a large replicase polyprotein, a spike (S) protein, an alphacoronavirus-specific accessory membrane protein, an envelope protein, a membrane protein, and a nucleocapsid (N) protein.

**Table:** Sequencing results of porcine epidemic diarrhea virus isolated from pigs in Germany, 2014*

Isolate	Mapped reads	Average depth	Total length	GenBank accession no.	Variant position (full genome)	Variant position (in CDS)	Codon change	Amino acid substitution	Variant frequency, %
L00719	30,723	146	28,028	LM645058	19,334	G19042U polyprotein	agu→auu	S6348I	48
					20,880	U247G S protein	ucu→gcu	S83A†	41
					21,017	U384A S protein	aau→aaa	N128K‡	43
					21,232	C599U S protein	gcu→guu	A200V§	14
					21,328	G695U S protein	agu→auu	S232I¶	41
					22,527	C1894U S protein	cuu→uuu	L632F#	13
					22,844	C2211U S protein	gac→gat	Silent	46
					22,970	U2337C S protein	auu→auc	Silent	44
L00721	19,274	104	28,028	LM645057	None	None	None	None	None

*CDS, coding sequences; S protein, spike protein. Blank cells indicate not applicable. †aa 83A also found in strain OH851 (GenBank accession no. KJ399978). ‡aa 128K found in all compared reference sequences. §aa 200V not detected in reference sequences. ¶aa 232I detected in recent highly virulent strains (GenBank accession nos. KJ778615, KF272920, KF468753, KJ584361, KJ408801, KF650374, KF468752, KF267450, KC210147, JN825712, KC210145). #aa 632F detected in reference strain KF267450 only.

Comparative analyses of the full genomes showed that the strains share a very high (99.5%) identity with the new variant OH851 (GenBank accession no. KJ399978) recently reported from the United States (*6*). A more comprehensive comparison of 21 full-length PEDV genomes from different years and locations revealed lower similarities (≈98.7%) with currently circulating highly virulent strains from the United States and from China (Technical Appendix Figure, panel A). In contrast, the new isolates from Germany, PEDV/GER/L00719/2014 and PEDV/GER/L00721/2014, are less similar (97.1%) to the isolate from Europe, CV777, which dates back to the 1970s (*12*).

The nucleotide alignment of the obtained PEDV genomes and the available references from the database revealed a region with high variability of the first 1,200 nt in the 5′ portion of the S protein–coding sequences (Technical Appendix Figure, panel B). The N terminal S1 domain of the coronavirus S protein is necessary for virus attachment by interaction with host cell receptors (*13*) and might therefore be highly mutable.

Although in-depth analysis of the deep-sequencing data for PEDV/GER/L00721/2014 revealed a genetically homogenous population, this analysis for PEDV/GER/L00719/2014 uncovered a mixed viral population with a total of 8 single-nucleotide variants. One nonsynonymous single-nucleotide variant (variant position G19042U, amino acid substitution S6348I) was detected in the polyprotein coding sequences. Of note, 7 single-nucleotide variants are located in the aforementioned variable region in the S protein coding sequences, 5 of which are nonsynonymous (Table), thereby confirming the high variability in the N terminal part of the S protein.

**Figure 2:**
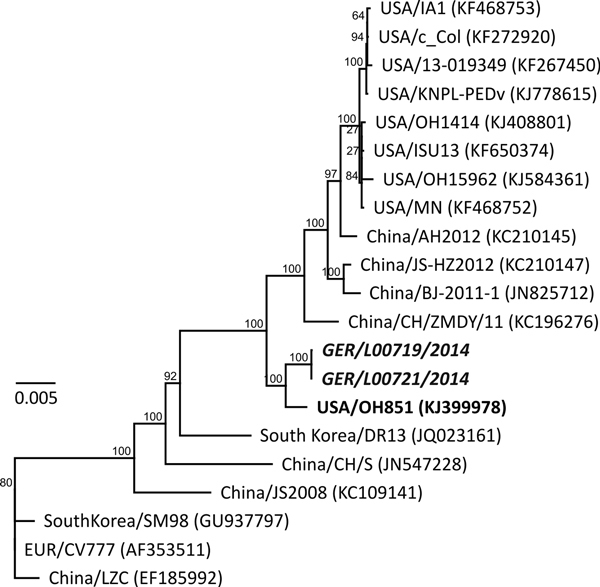
Phylogenetic analysis based on 21 full-length porcine epidemic diarrhea virus (PEDV) genomes. The new strains from Germany (PEDV/GER/L00719/2014 and PEDV/GER/L00721/2014, in boldface and italics) and the new 2014 PEDV variant from the United States (OH851, in boldface) were included and compared with current circulating strains from the United States and China. The tree was constructed by using PhyML (*11*). Numbers above branches indicate proportions calculated from 1,000 bootstrap replicates: The scale bar represents nucleotide substitutions per site.

Because quite extensive differences (≈50 aa) were found between the recent N terminal S protein region of the isolates from Germany and the highly virulent PEDV strains from the United States and China, the isolates from Germany described in this article seem to not be directly linked to the highly virulent PEDV strains circulating in the United States (Figure 2). In contrast, the recent isolates from Germany and strain OH851 share not only high identity over the entire genome, including the highly variable 5′ region of the S protein coding sequences, but also their clinical phenotype observed under field conditions.

## Conclusions

PEDV infection was confirmed in a pig herd in Germany in 2014. Comparative analyses of full-length sequences revealed that the isolates from these pigs in Germany show very high nucleotide similarity with strain OH851 found in the United States in 2014. However, differences exist that distinguish the strains from Germany from the highly virulent PEDV strains that caused the major losses in the United States. Given the fact that PEDV surveillance has been lacking in Germany, we cannot exclude the possibility that the strains described here have already been circulating in Europe for a longer time or were indeed recently introduced from the United States or Asia to Europe. Therefore, our report provides useful information about recent PEDV strains in Europe, but a comprehensive evaluation is still difficult because of a lack of data about additional strains. Future studies should therefore concentrate on analysis of additional PEDV from different years and locations.

Technical Appendix Comparison of 21 porcine epidemic diarrhea virus genomes from different years and locations. Pairwise similarity matrix based on full-length genomes and schematic representation of the nucleotide sequence alignment of the complete spike protein coding sequences.
