# Supplementary material for: Comparison of Porcine Epidemic Diarrhea Viruses from Germany and the United States, 2014
Source: Emerg Infect Dis. 2015 Mar;21(3):493–6. doi: 10.3201/eid2103.141165 (PMC4344272; doi:10.3201/eid2103.141165)
Supplement: Technical Appendix — Comparison of 21 porcine epidemic diarrhea virus genomes from different years and locations. Pairwise similarity matrix based on full-length genomes and schematic representation of the nucleotide sequence alignment of the complete spike protein coding sequences. [file 14-1165-Techapp-s1.pdf]

Article DOI: http://dx.doi.org/10.3201/eid2103.141165

# Comparison of Porcine Epidemic Diarrhea Viruses from Germany and United States, 2014

## Technical Appendix

**A**

| | *GER/L00719/2014* | *GER/L00721/2014* | **KJ399978** | KC196276 | KJ778615 | KF272920 | KF468753 | KF267450 | KJ584361 | KJ408801 | KF650374 | KF468752 | KC210145 | KC210147 | JN825712 | JQ023161 | JN547228 | KC109141 | AF353511 | EF185992 | GU937797 |
|---|---|---|---|---|---|---|---|---|---|---|---|---|---|---|---|---|---|---|---|---|---|
| *GER/L00719/2014* | | 100,0% | 99,5% | 98,9% | 98,7% | 98,7% | 98,7% | 98,7% | 98,7% | 98,6% | 98,6% | 98,6% | 98,5% | 98,6% | 98,5% | 97,9% | 97,6% | 97,3% | 97,1% | 96,8% | 96,6% |
| *GER/L00721/2014* | 100,0% | | 99,5% | 98,9% | 98,7% | 98,7% | 98,7% | 98,7% | 98,7% | 98,6% | 98,6% | 98,6% | 98,5% | 98,6% | 98,5% | 97,9% | 97,6% | 97,3% | 97,1% | 96,8% | 96,6% |
| **USA/OH851 (KJ399978)** | 99,5% | 99,5% | | 98,8% | 99,1% | 99,1% | 99,1% | 99,1% | 98,9% | 99,0% | 99,0% | 99,0% | 98,7% | 98,7% | 98,7% | 97,9% | 97,6% | 97,4% | 97,1% | 96,8% | 96,6% |
| China/CH/ZMDY/11 (KC196276) | 98,9% | 98,9% | 98,8% | | 99,0% | 99,0% | 99,0% | 99,0% | 99,1% | 99,1% | 99,1% | 99,1% | 99,1% | 99,1% | 99,1% | 97,6% | 97,2% | 97,0% | 96,8% | 96,5% | 96,3% |
| USA/KNPL-PEDv (KJ778615) | 98,7% | 98,7% | 99,1% | 99,0% | | 100,0% | 100,0% | 99,9% | 99,8% | 99,8% | 99,9% | 99,9% | 99,5% | 99,4% | 99,3% | 97,7% | 97,3% | 97,0% | 96,8% | 96,5% | 96,3% |
| USA/c_Col (KF272920) | 98,7% | 98,7% | 99,1% | 99,0% | 100,0% | | 100,0% | 99,9% | 99,7% | 99,8% | 99,9% | 99,9% | 99,5% | 99,4% | 99,3% | 97,6% | 97,3% | 97,0% | 96,8% | 96,5% | 96,3% |
| USA/IA1 (KF468753) | 98,7% | 98,7% | 99,1% | 99,0% | 100,0% | 100,0% | | 99,9% | 99,7% | 99,8% | 99,8% | 99,8% | 99,5% | 99,4% | 99,3% | 97,6% | 97,3% | 97,0% | 96,7% | 96,5% | 96,3% |
| USA/13-019349 (KF267450) | 98,7% | 98,7% | 99,1% | 99,0% | 99,9% | 99,9% | 99,9% | | 99,7% | 99,8% | 99,8% | 99,8% | 99,5% | 99,3% | 99,3% | 97,6% | 97,3% | 97,0% | 96,7% | 96,5% | 96,3% |
| USA/OH15962 (KJ584361) | 98,7% | 98,7% | 98,9% | 99,1% | 99,8% | 99,7% | 99,7% | 99,7% | | 99,8% | 99,9% | 99,9% | 99,5% | 99,3% | 99,2% | 97,7% | 97,3% | 97,0% | 96,8% | 96,5% | 96,3% |
| USA/OH1414 (KJ408801) | 98,6% | 98,6% | 99,0% | 99,1% | 99,8% | 99,8% | 99,8% | 99,8% | 99,8% | | 99,9% | 99,9% | 99,6% | 99,3% | 99,3% | 97,7% | 97,3% | 97,1% | 96,8% | 96,5% | 96,3% |
| USA/ISU13 (KF650374) | 98,6% | 98,6% | 99,0% | 99,1% | 99,9% | 99,9% | 99,8% | 99,8% | 99,9% | 99,9% | | 100,0% | 99,6% | 99,4% | 99,3% | 97,7% | 97,3% | 97,0% | 96,8% | 96,5% | 96,3% |
| USA/MN (KF468752) | 98,6% | 98,6% | 99,0% | 99,1% | 99,9% | 99,9% | 99,8% | 99,8% | 99,9% | 99,9% | 100,0% | | 99,6% | 99,4% | 99,3% | 97,7% | 97,3% | 97,1% | 96,8% | 96,5% | 96,3% |
| China/AH2012 (KC210145) | 98,5% | 98,5% | 98,7% | 99,1% | 99,5% | 99,5% | 99,5% | 99,5% | 99,5% | 99,6% | 99,6% | 99,6% | | 99,5% | 99,4% | 97,7% | 97,3% | 97,1% | 96,8% | 96,6% | 96,3% |
| China/JS-HZ2012 (KC210147) | 98,6% | 98,6% | 98,7% | 99,1% | 99,4% | 99,4% | 99,4% | 99,3% | 99,3% | 99,3% | 99,4% | 99,4% | 99,5% | | 99,7% | 97,8% | 97,4% | 97,2% | 96,9% | 96,6% | 96,4% |
| China/BJ-2011-1 (JN825712) | 98,5% | 98,5% | 98,7% | 99,1% | 99,3% | 99,3% | 99,3% | 99,3% | 99,2% | 99,3% | 99,3% | 99,3% | 99,4% | 99,7% | | 97,8% | 97,3% | 97,1% | 96,9% | 96,7% | 96,5% |
| SouthKorea/DR13 (JQ023161) | 97,9% | 97,9% | 97,9% | 97,6% | 97,7% | 97,6% | 97,6% | 97,6% | 97,7% | 97,7% | 97,7% | 97,7% | 97,7% | 97,8% | 97,8% | | 98,1% | 97,8% | 97,7% | 97,4% | 97,2% |
| China/CH/S (JN547228) | 97,6% | 97,6% | 97,6% | 97,2% | 97,3% | 97,3% | 97,3% | 97,3% | 97,3% | 97,3% | 97,3% | 97,3% | 97,3% | 97,4% | 97,3% | 98,1% | | 97,5% | 97,4% | 97,1% | 96,9% |
| China/JS2008 (KC109141) | 97,3% | 97,3% | 97,4% | 97,0% | 97,0% | 97,0% | 97,0% | 97,0% | 97,0% | 97,1% | 97,0% | 97,1% | 97,1% | 97,2% | 97,1% | 97,8% | 97,5% | | 97,7% | 97,4% | 97,2% |
| EUR/CV777 (AF353511) | 97,1% | 97,1% | 97,1% | 96,8% | 96,8% | 96,8% | 96,7% | 96,7% | 96,8% | 96,8% | 96,8% | 96,8% | 96,8% | 96,9% | 96,9% | 97,7% | 97,4% | 97,7% | | 99,7% | 99,5% |
| China/LZC (EF185992) | 96,8% | 96,8% | 96,8% | 96,5% | 96,5% | 96,5% | 96,5% | 96,5% | 96,5% | 96,5% | 96,5% | 96,5% | 96,6% | 96,6% | 96,7% | 97,4% | 97,1% | 97,4% | 99,7% | | 99,2% |
| SouthKorea/SM98 (GU937797) | 96,6% | 96,6% | 96,6% | 96,3% | 96,3% | 96,3% | 96,3% | 96,3% | 96,3% | 96,3% | 96,3% | 96,3% | 96,3% | 96,4% | 96,5% | 97,2% | 96,9% | 97,2% | 99,5% | 99,2% | |

current isolates

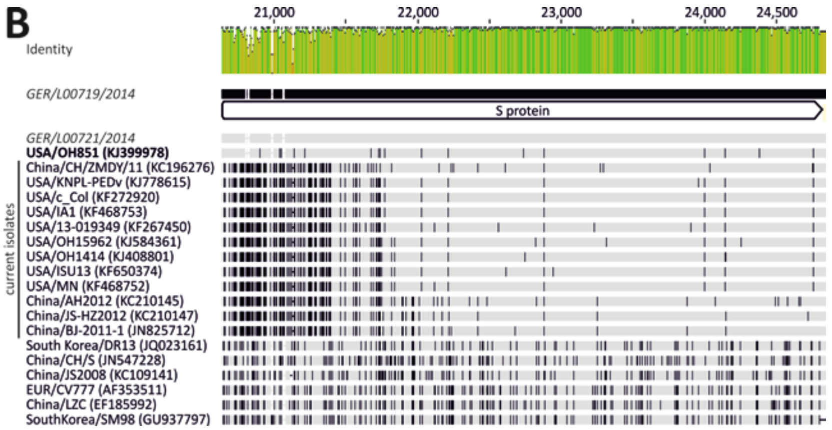


Appendix Figure. Nucleotide sequence alignment (MAFFT) (*1*) of whole-genome sequences of 21 isolates of porcine epidemic diarrhea virus (PEDV). The new strains PEDV/GER/L00719/2014 and PEDV/GER/L00721/2014 (italics) from Germany and the new 2014 PEDV US variant OH851 (boldface) were included and compared with current circulating strains from the United States and China (current isolates). A) Pairwise similarity matrix based on full-length genomes. Similarity is given in percent identity.

B) Schematic representation of the nucleotide sequence alignment of the complete spike protein coding sequences to depict the variable region in the 5′ part. Sequence identity is given in the upper color-coded graph (dark green, high similarity; light green, lower similarity; red, low similarity). Nucleotide positions that differ relative to the recent German strains are marked in black.

**Reference**

1. Katoh K, Misawa K, Kuma K, Miyata T. MAFFT: a novel method for rapid multiple sequence alignment based on fast Fourier transform. Nucleic Acids Res. 2002;30:3059–66. PubMed http://dx.doi.org/10.1093/nar/gkf436
